# Effect of Microfibrillated Cellulose on Microstructure and Properties of Poly(vinyl alcohol) Foams

**DOI:** 10.3390/polym10080813

**Published:** 2018-07-24

**Authors:** Gennaro Gentile, Mariacristina Cocca, Roberto Avolio, Maria Emanuela Errico, Maurizio Avella

**Affiliations:** Institute for Polymers, Composites and Biomaterials, Italian National Research Council-Via Campi Flegrei 34, 80078 Pozzuoli, NA, Italy; gennaro.gentile@ipcb.cnr.it (G.G.); roberto.avolio@ipcb.cnr.it (R.A.); mariaemanuela.errico@ipcb.cnr.it (M.E.E.); maurizio.avella@ipcb.cnr.it (M.A.)

**Keywords:** microfibrillated cellulose, poly(vinyl alcohol), foams, microstructure, properties

## Abstract

Poly(vinyl alcohol) foams, containing different amounts of microfibrillated cellulose, were prepared through an eco-friendly procedure based on high-speed mixing and freeze-drying. The effect of filler amount on cell shape and regularity was studied by scanning electron microscopy (SEM) and the evolution of the microstructure was assessed through dynamic cryo-SEM. Fourier Transformed Infrared Analysis and Differential Scanning Calorimetry measurements revealed the presence of hydrogen bond interaction among cellulosic filler and the matrix. The modulus and compression deflection of neat PVA were significantly improved by increasing the amount of microfibrillated cellulose content with respect to foams realised with pulp cellulose fibers.

## 1. Introduction

Polymer foams, due to their unique structure and properties, are widely used in all those applications for which light-weight, thermal/acoustic barrier effects and shock-impact absorption are required, ranging from the automotive sector to packaging systems, from construction to technical footwear [1]. Nevertheless, petroleum-based polymer foams possess serious environmental problems after end-use, due to their non-biodegradability and difficult disposal. In this context, there is a strong motivation to replace petroleum-based polymers with polymers from renewable resources [2,3] and biodegradable polymer [4].

Poly(vinyl alcohol) (PVA), a vinyl water-soluble polymer susceptible to biodegradation in the presence of suitable microorganisms [5], is of great interest in realizing environmentally compatible materials for a wide range of applications. Due to the unique non-toxicity, biocompatibility and biodegradability, PVA has been widely used in the production of papers, pharmaceuticals, cosmetics, and food industries [6,7]. An attractive way to improve the mechanical performances of PVA, preserving its biodegradability, is the use of cellulose or nanocellulose as a reinforcing phase [8].

Pulp cellulose fibres are widely used as filler in polymer composites [9,10]. In recent years, microfibrillated cellulose (MFC), which consists of mechanically fibrillated pulp into nano- to submicron fibres, forming a web-like network, has received significant research attention, also for the realisation of composites, because of its high aspect ratio, high stiffness, strength, and biodegradability [11,12,13,14,15,16,17].

In previous works [18,19], the preparation of PVA foams through an eco-sustainable methodology based on high speed mixing, without using chemical blowing agents, has been reported. The effect of low amounts (up to 5 wt %) of MFC on the properties of PVA-based foams was investigated [19], revealing a dual-pore microstructure and an improvement of the mechanical properties of the foams. unclear. In this work, high MFC content (up to 40 wt %) PVA foams were prepared and characterised with the aim of understanding and clarifying the effect of the addition of a high amount of filler on the properties and morphologies of the PVA foams. The mechanism of formation of the double porosity was clarified and the effect of MFC on the properties of the foams was analysed through physico-mechanical characterisations in comparison to PVA foams containing pulp cellulose fibres.

## 2. Experimental

### 2.1. Materials

Microfibrillated cellulose (MFC, Celish FD-100 G) was kindly supplied by Daicel Chemical Industries, Ltd., (Tokyo, Japan). MFC water slurry containing 10 wt % fibres was prepared from highly refined pure plant fibre materials treated in a super-high-pressure homogenizer. MFC fibre diameter ranges were between 0.01 μm and 10 μm.

Highly pure wood pulp cellulose, WPC, trade name Arbocel Bc1000, was supplied by JRS PharmaGmbH (Rosenberg, Germany). It was dried under vacuum at 90 °C for 24 h before use. The average length and diameter of the WPC fibres were 700 μm and 20 μm, respectively.

Partially hydrolysed (87–90%) poly(vinyl alcohol), with weight-average molecular weight (*M_w_*) in the range of 30,000–70,000, and glycerol (GLY) were purchased from Sigma-Aldrich (Milan, Italy).

### 2.2. Foam Preparation

Poly(vinyl alcohol) was dispersed in deionised water (0.08 g/mL), and glycerol, 30 wt % with respect to poly(vinyl alcohol), was added as plasticizer. The mixture was heated at 80 °C under stirring for about 2 h and the obtained solution was then cooled at room temperature. Different amounts of MFC were added to the solutions in order to reach a final solid content of MFC equal to 5, 10, 30 and 40 wt % with respect to the weight of poly(vinyl alcohol). The obtained suspensions were stirred for 1 h at room temperature, then poured into a high-speed mixer, where they were kept for 2 min at high stirring rate (about 3000 rpm), following the procedure optimised in a previous work [18]. The foamed suspensions were cast in cylindrical moulds (40 mm diameter and 30 mm height), frozen in liquid nitrogen, then kept for 24 h at −18 °C and finally lyophilised at T −80 °C and P 5·10^−2^ torr using a freeze dryer apparatus. After this procedure, the foams were completely dried.

The same preparation method was used to obtain neat poly(vinyl alcohol)/glycerol foams. The resulting foam samples were coded as PVA, PVA-MFC5, PVA-MFC10, PVA-MFC30 and PVA-MFC40. Poly(vinyl alcohol)-based foam containing 40 wt % of dried WPC fibres was coded PVA-WPC40.

### 2.3. Methods

Morphological analysis of cellulose fibres and cryogenically fractured PVA foams was performed using a FEI Quanta 200 FEG scanning electron microscope, SEM, Thermo fischer Scientific Hillsboro, OR, USA) in high vacuum mode, using an accelerating voltage ranging between 15 and 20 kV and a secondary electron detector (Everhart-Thornley detector). Before the analysis, dried specimens were mounted on aluminium stubs by means of carbon adhesive disks and coated with a thin layer (about 15 nm thick) of an Au-Pd alloy by means of a Baltec MED 020 Sputter Coater System (Pfäffikon, Switzerland).

Dynamic cryo-SEM experiments were carried out by means of a cryo-system Gatan Alto 1000E (Pleasanton, CA, USA) installed on the FEI Quanta 200 FEG SEM in order to evaluate the process of formation of the foams. A small amount of foamed suspension was placed on the cryo-SEM holder, mounted on the cryo-transfer rod, slam-frozen in nitrogen slush and transferred to the cryo-chamber, where it was cryo-fractured and sputter-coated with gold/palladium. The sample was finally moved to the SEM chamber, where the fractured surface was observed at −140 °C, using an acceleration voltage of 5–10 kV and a secondary electron detector. Subsequently, the frozen suspension was removed from the SEM chamber, heated up to −80 °C at 10^−4^ torr and inserted again into the SEM chamber for observation of the obtained foamed morphology using an acceleration voltage of 5–10 kV and a secondary electron detector.

Fourier Transform Infrared, FTIR, spectra of the samples were recorded at room temperature by means of a Perkin Elmer Spectrum 100 FTIR spectrometer (Waltham, MA, USA), equipped with an attenuated total reflectance accessory (ATR). The scanned wavenumber range was 4000–650 cm^−1^. All spectra were recorded at a resolution of 4 cm^−1^, and 16 scans were averaged for each sample.

Thermal properties of foams were investigated using a Mettler DSC 822e calorimeter, Mettler-Toledo, Inc. (Columbus, OH, USA), equipped with a liquid nitrogen accessory for fast cooling. The calorimeter was calibrated in temperature and energy using indium. Dry nitrogen was used as purge gas at a rate of 30 mL/min during the measurement. A small piece of sample, weighing about 10 mg, was encapsulated in standard aluminium 40 μL pans. Samples were heated from 25 °C to 250 °C at a rate of 10 °C/min and held at 250 °C for 2 min to eliminate any prior thermal history. Then, they were cooled to −70 °C at a rate of 10 °C/min, and finally the samples were reheated to 250 °C at a heating rate of 10 °C/min. DSC curves were analysed using the STARe Software 8.1, Mettler-Toledo GmbH (Columbus, OH, USA).

Compression tests of the foams were performed with an Instron Universal Testing Machine, Model 4505, (Norwood, MA, USA) according to the standard test method D624. Prior to testing, the specimens were conditioned at 50% RH and 25 °C for 24 h. Cylinder-shaped foams were used in the compression measurements, placed vertically within the compression plates, and the crosshead speed was set at 12.5 mm/min. Compression deflection (CD) was calculated as the ratio of the load at 25% deflection and the area of the tested sample. For each sample, 5 specimens were tested, and the average value was reported. 

The deformation mechanism of the foams under compression was evaluated by means of compression tests coupled to SEM analysis, using a Deben microtensile/compression stage equipped with a 20 N cell load (Suffolk, UK). Compression tests were performed on small cubic specimens (about 30 mm^3^) within the SEM chamber, imaging the sample PVA-MFC10 during the test in low-vacuum mode (P_H2O_ = 0.75 torr) at 30 kV using a large field detector.

The water vapour absorption behaviour of PVA-based foams, dried MFC and WPC fibres was investigated through a gravimetric determination of the swelling ratio (SR), calculated using the equation:

(1) $$\mathrm{SR}=100\times \frac{W_{s}-W_{d}}{W_{d}}$$

where *W_s_* is the weight of the sample in the swollen state and *W_d_* is the weight of the dried sample. 

To measure the swelling ratio, pre-weighed dried samples were placed in a climatic chamber at 25 °C and 50% RH, and the weight of the swollen samples was measured after 0.25, 0.5, 1, 2, 4, 6 and 24 h. For each sample, 5 specimens were tested, and the average value was reported.

## 3. Results and Discussion

### 3.1. Foams Microstructure

**Figure 1:**
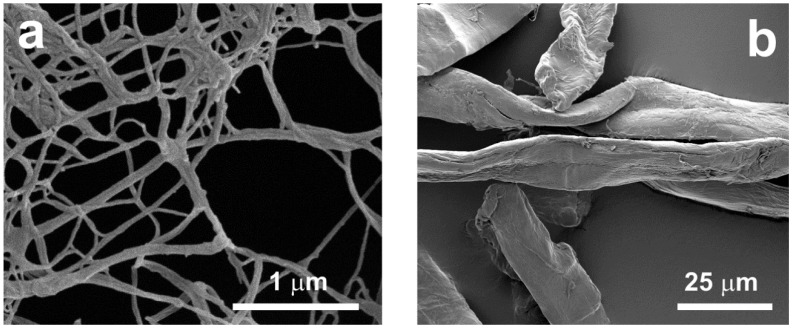
SEM images of: (**a**) MFC; (**b**) WPC.

Scanning electron micrographs of MFC and WPC are reported in Figure 1. Figure 1a clearly evidences that MFC consists of cellulose microfibrils with diameters lower than 100 nm, forming a network, and bundles of these microfibrils. Nevertheless, MFC contains a certain number of larger fibre fragments and unfibrillated fibres, characterised by micrometric dimensions. Morphological features of WPC are shown in Figure 1b. The average fibre diameter is about 15 μm, and the average length of the commercial WPC is 700 μm [20].

As detailed in the experimental section, PVA foams were realised by using a high-speed mixer. In particular, MFC and WPC were dispersed in PVA water solutions containing glycerol. Foaming was performed at high stirring rates; after that, the foamed suspensions were cast in moulds, frozen in liquid nitrogen, and finally lyophilised.

**Figure 2:**
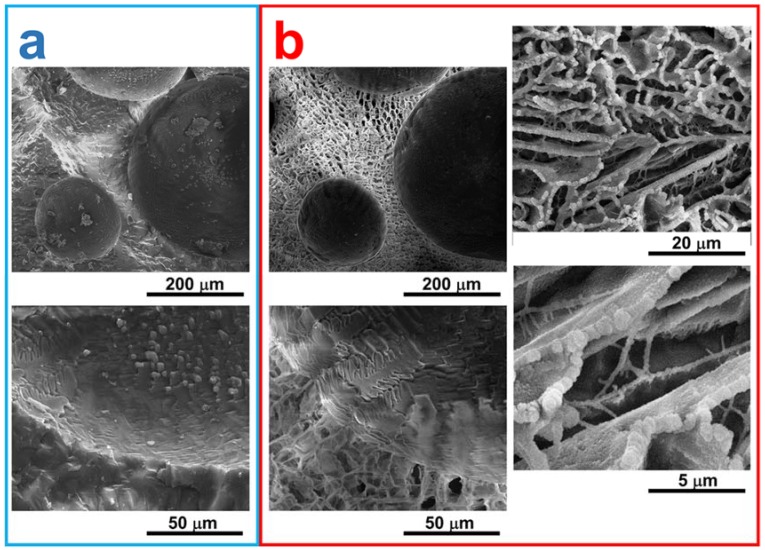
Cryo-SEM images of the sample PVA-MFC5: (**a**) Sample frozen in liquid nitrogen and observed at −140 °C; (**b**) Same areas of the sample after water sublimation at −80 °C.

In order to investigate the formation of the porous structure in PVA-based foams, the water sublimation process from the frozen PVA sample containing 10 wt % of MFC was reproduced in the SEM chamber by a dynamic cryo-SEM experiment. Results are shown in Figure 2.

As can be observed in Figure 2a, when the foamed solution of PVA containing cellulose is frozen at the liquid nitrogen temperature, spherical pores due to air entrapped during the foaming process are present, with variable diameters ranging between 100 and 300 μm, while no porous structures are detectable on the cell walls. In fact, before lyophilisation, dense walls can be detected, constituted by the frozen water dispersion of PVA and cellulose fibres. From Figure 2b, it can be observed that the lyophilisation process does not affect the size and shape of the large spherical pores derived from air entrapment. On the contrary, as shown in the micrographs at higher magnification, lyophilisation induces the formation of another type of porosity within the foam walls, due to water sublimation. The resulting pores, with sizes ranging between 2 and 10 μm and irregular in shape, are interconnected, and this structure joins adjacent spherical pores derived from the foaming process. The resulting foam microstructure is therefore constituted by a double interconnected porosity deriving from the foaming process (large spherical pores) and from the water sublimation (small irregular pores).

**Figure 3:**
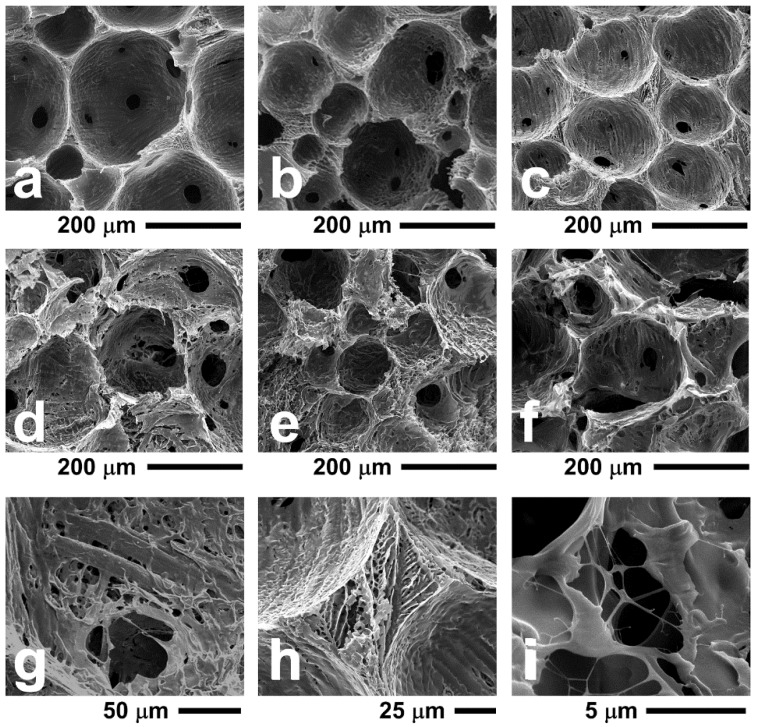
SEM micrographs of: (**a**) neat PVA foam; (**b**) PVA-MFC5; (**c**) PVA-MFC10; (**d**) PVA-MFC30; (**e**,**g**–**i**) PVA-MFC40; (**f**) PVA-WPC40.

SEM images of cryogenically fractured foamed samples containing different amounts of MFC and WPC are reported in Figure 3.

Neat PVA foams, as well as foams containing MFC or WPC, are characterised by a double porosity, with the presence of large spherical pores due to air entrapped during the foaming process and by small irregular pores due to water removal occurring during lyophilisation. As shown in a previous work [19], by addition of a small amount of MFC (1–5 wt %) a progressive decrease of the average diameter of the large pores and an increase of the cell density is obtained. The average diameter of the PVA foam cells is about 190 μm, and the cell density is about 20 cells/mm^2^, whereas adding 5 wt % of MFC, the foam shows an average diameter of about 105 μm and a cell density of about 60 cells/mm^2^. A further increase of the amount of MFC up to 10 wt % (Figure 3c) does not induce further significant changes in the average cell diameter or of the cell density with respect to that recorded for PVA-MFC5. Moreover, at 30 or 40 wt % of MFC loading (Figure 3d,e, respectively), the obtained foams are characterised by an irregular cell structure. This phenomenon is also observed for foam containing 40 wt % of WPC (see, for instance, Figure 3f), and this is attributed to the high fibre content, which hinders a regular cell expansion during foaming. This effect is confirmed by the SEM image in Figure 3g (PVA-MFC30), where it is shown that the cellulose fibres with large diameters (about 10 μm) present in MFC are located within the cell walls, thus acting as irregularities that can hinder the formation of regular spherical pores during foaming.

Smaller pores due to water removal during lyophilisation are shown in Figure 3h, where a detail of the cell walls at the intersection of four large spherical pores is shown. Moreover, the presence of nanometer-sized MFC fibres located within the walls of the foam are shown in Figure 3i for the sample PVA-MFC30. MFC nanofibres show a good adhesion with the PVA phase, creating a fine interconnected structure within the smaller pores, thus leading to the hypothesis of a significant reinforcing effect of MFC of the foams.

### 3.2. FTIR Analysis

**Figure 4:**
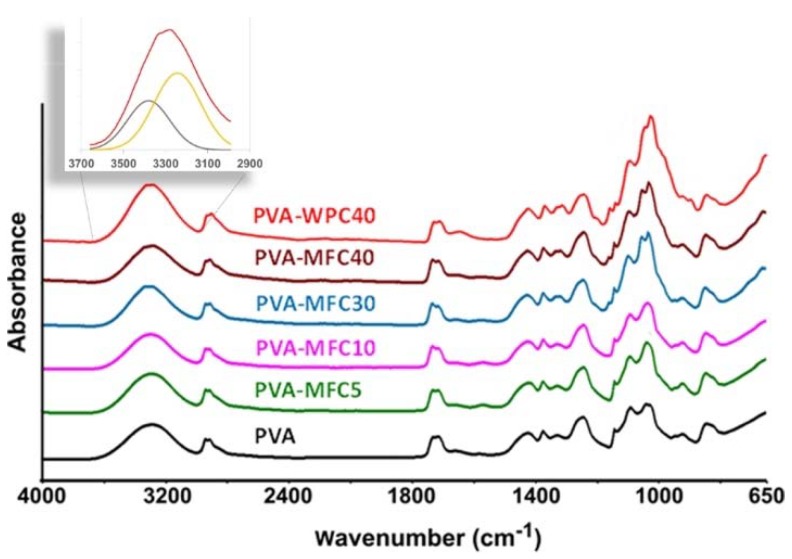
FTIR spectra of PVA, PVA-MFC and PVA-WPC foams. In the inset, an example of curve fit is reported.

The interaction of PVA and cellulose in the cell walls of both PVA-MFC and PVA-WPC foams was evaluated by FTIR spectroscopy from the analysis of the O–H stretching vibration band. FTIR spectra are reported in Figure 4.

FTIR spectrum of neat PVA exhibits absorption bands related to hydroxyl and acetate groups. The large band observed between 3700 and 2980 cm^−1^ is due to the stretching of the O–H group; the band observed in the range 2980–2800 cm^−1^ corresponds to the stretching C–H from the alkyl groups; and the peaks between 1770–1680 cm^−1^ are assigned to the C=O and C–O stretching from residual acetate groups in the PVA matrix.

For both PVA-MFC and PVA-WPC foams, FTIR spectra showed the typical bands corresponding to the polymer matrix. The presence of low amounts of MFC does not significantly alter the spectrum of PVA and in particular the shape of O–H stretching band. At higher amounts of MFC, the increased presence of the cellulose is evidenced by the gradual increase of absorption bands centred at 1024 and 1052 cm^−1^ is due to C–C, C–OH, C–H ring and side group vibrations [21]. Moreover, at high cellulose amounts, the band typical of O–H stretching is split into two components centred at around 3340 and 3270 cm^−1^, as illustrated in the inset of Figure 4, which are typical of the stretching O−H from the intermolecular and intramolecular hydrogen bonds. This phenomenon is explained by the strong interactions present, mainly due to hydrogen bonding between the hydroxyl groups in the polymer chains with the hydroxyl groups located on cellulose fibres. This is similar to what has already been reported for fibrous PVA/cellulose nanocrystal composites [21], and for PVA/chitin [22] and PVA/chitosan systems [23]. This effect was also observed in the PVA-WPC40 sample, confirming that it is relevant at high cellulose contents, independently from the cellulose aspect ratio.

### 3.3. Thermal Analysis

**Figure 5:**
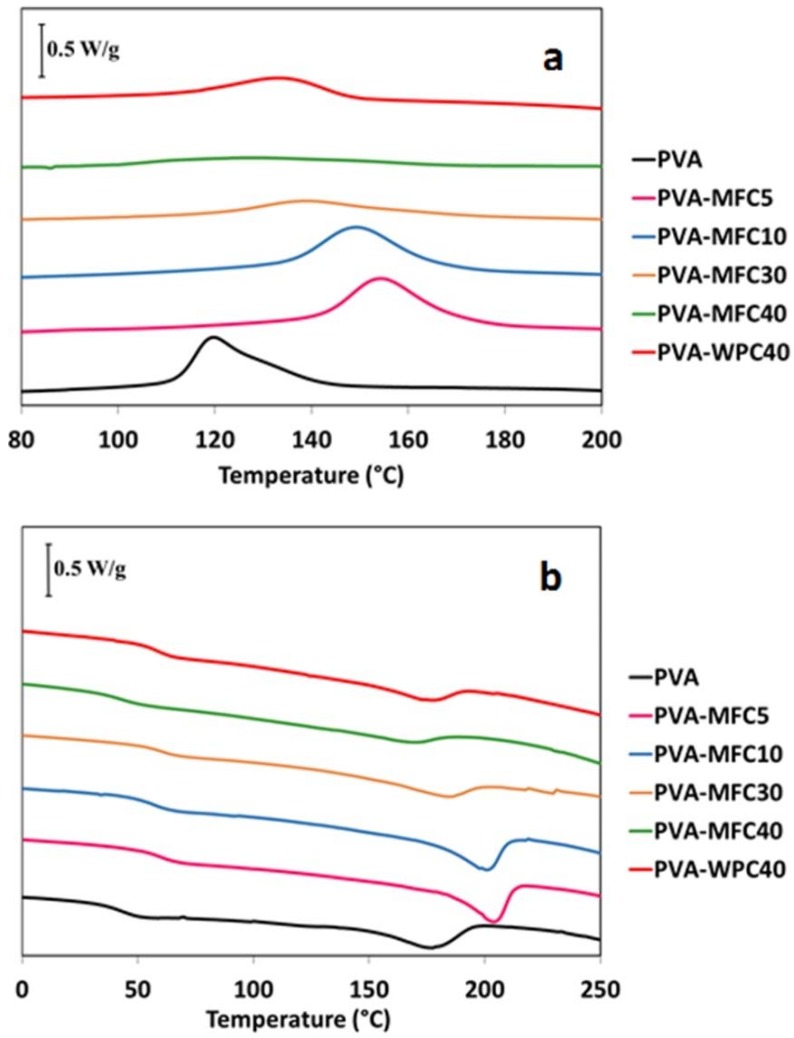
DSC curves of PVA-based foams. (**a**) cooling cycle, and (**b**) second heating cycle.

The thermal properties of foams were determined by means of DSC analysis. The first heating cycle showed pronounced exothermic peaks due to the water evaporation for all the samples. Therefore, in Figure 5, only the cooling and the second heating cycles of PVA and PVA-MFC foams are reported, in which the moisture effect is not present.

The addition of MFC increases the crystallisation temperature (*T*_c_) of neat PVA. In particular, at 5 wt % MFC loading, a 35 °C rise in temperature with respect to neat PVA was recorded. Further increase of MFC up to 40 wt % induces a progressive decrease of the crystallisation temperature with respect to that recorded for PVA-MFC5. This result could be explained by considering that at low contents, MFC acts as a nucleating agent, shifting *T*_c_ towards a higher temperature with respect to neat PVA. In contrast, at higher loadings, MFC can progressively inhibit PVA intermolecular interactions by hindering the formation of hydrogen bonds, leading to the progressive shift of *T_c_* towards lower temperatures [24].

The degree of crystallinity, reported in Table 1, was calculated based on Equation (2):

(2) $$X_{c}=\frac{\Delta H_{m}}{w\cdot \Delta H_{m}^{0}}$$

where *w* is the weight fraction of PVA in the foams, Δ*H*_m_ is the heat of fusion, and $\Delta H_{m}^{0}$ is the heat of fusion of a 100% crystalline PVA, 161.6 J/g [25].

The trend of crystallinity is similar to that observed for the crystallisation temperature. *X_c_* reached the maximum value of 19.4% for PVA-MFC5, and then gradually decreased, starting from 10 wt % of MFC loading. This result confirms that, although MFC could act as a nucleating agent, at higher amounts, it affects the PVA chain interactions, decreasing the overall crystallinity of the foam. A similar effect is observed in the presence of 40 wt % of WPC, with PVA-WPC40 showing a decrease in the crystallinity of PVA.

Moreover, MFC and WPC also affect the glass transition temperature, *T*_g_, and the melting temperature of neat PVA foam, see Figure 5b and Table 1. *T*_g_ is found to significantly increase with an increase in the amount of MFC or WPC. This phenomenon can be ascribed to the formation of strong interactions between PVA and cellulose fibres that, reducing the molecular mobility of amorphous PVA chains at the interface, induce a shift of *T*_g_ towards higher temperatures. Comparing the effects recorded for MFC and WPC at the same loadings, 40 wt %, the increase of *T*_g_ is more pronounced for the microfibrillated cellulose, characterised by a higher aspect ratio than WPC, thus indicating that the recorded difference is mainly due to the increased interfacial area between MFC and the polymer matrix.

Finally, with regard to the melting temperatures, *T*_m_ of PVA-based foams shows the same trend of *T*_c_ as a function of MFC and WPC amount, with a more pronounced increase at low filler loadings. Again, this is ascribed to strong interactions between PVA chain and cellulose fibres by hydrogen bonding forces. As a consequence, highly crystalline structures are formed at low amounts of MFC, while an inverse trend was obtained with higher amounts of MFC, restricting the capability of the matrix chains to form crystalline domains. At 40 wt % of cellulose loading, only small differences were recorded for MFC and WPC with respect to neat PVA.

### 3.4. Mechanical Analysis

**Figure 6:**
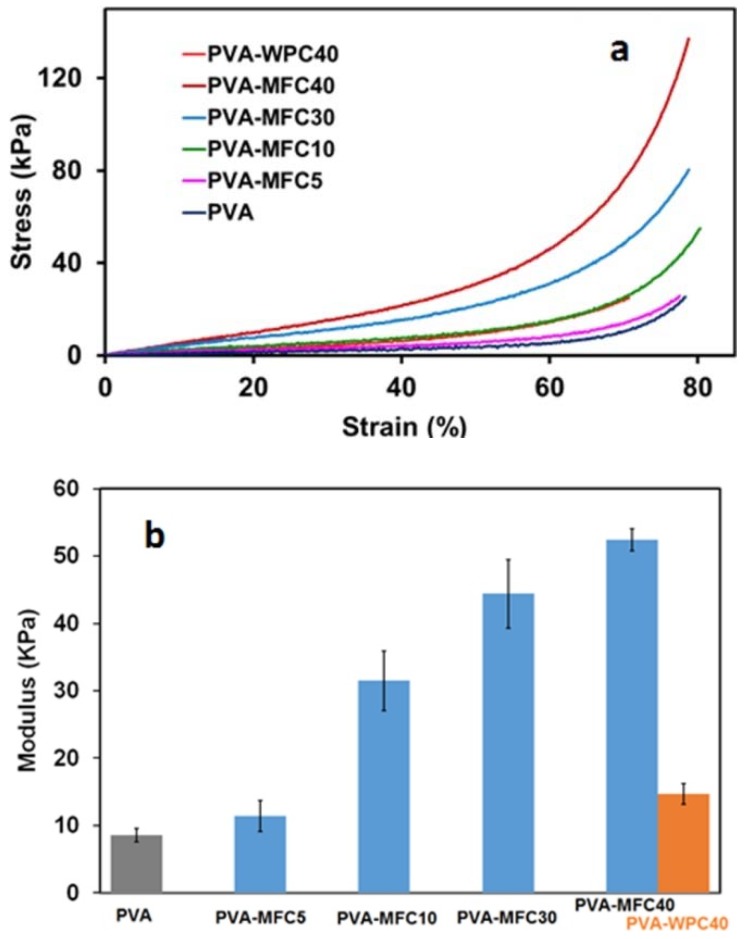

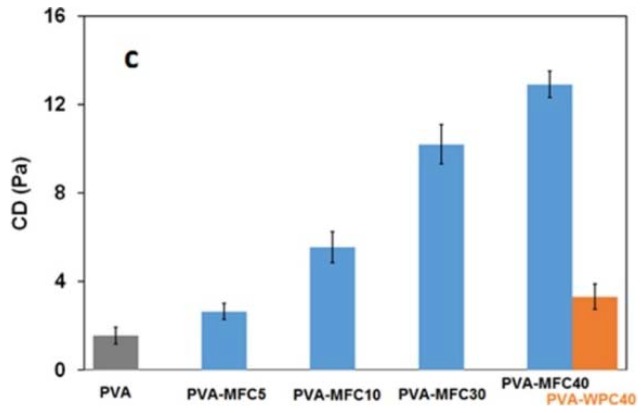
(**a**) Stress–strain curves; (**b**) compression moduli; and (**c**) compression deflection (CD) of PVA-based foams conditioned at 25 °C and 50% RH as a function of MFC and WPC amount.

In Figure 6a, the compressive stress–strain curves of foams at varying MFC contents and at 40 wt % of WPC loading are reported. For all the samples, at low strains, the compressive stress–strain curves show a linear elasticity step followed by a plateau-like region, in which there is a slow increase of the stress with increasing strain, which is followed due to the collapse of the cells. At higher strains, the densification regime starts, and stress rises steeply with strain.

The Young’s modulus and compression deflection reported in Figure 6b,c highlight that the addition of 5 wt % of MFC induces a rise in the compression moduli from values close to 8 ± 1 KPa of neat PVA foam to around 11 ± 1 KPa. Very significant increments of compression moduli were recorded with the addition of 10, 30 and 40 wt % of MFC. For these samples, moduli values of 31 ± 4 KPa, 44 ± 5 KPa and 52 ± 1 KPa were recorded, respectively. On the other hand, the addition of 40 wt % of WPC induced only a slight improvement of the compression modulus with respect to neat PVA foam, with values of around 15 ± 1 KPa. With a similar trend, the compression deflection of PVA-MFC foams significantly increases with the increase of MFC content, and also in this case, the effect is much more relevant for PVA-MFC foams, whereas WPC induced much smaller improvements.

**Figure 7:**
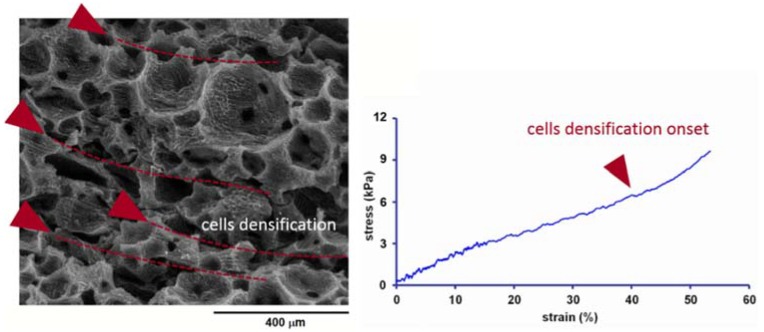
In-SEM compression test of the sample PVA-MFC-10, showing the mechanism of deformation of the foam.

An insight into the mechanism of cell deformation of the sample PVA-MFC10 was obtained by performing the compression test inside the SEM chamber. The results of this test are shown in Figure 7 (video available in the Supplementary Materials). The onset of the densification process is recorded at about 30% of deformation. In fact, at this strain, the collapse of the cells starts, with a progressive cell deformation responsible for the corresponding steep increase of the stress–strain curve, characteristic of the collapse region of the foam.

The considerable improvement of mechanical properties recorded for the samples containing a high amount of MFC could be due to a regular distribution of MFC, interacting well with the PVA matrix, in the cell wall, thus improving the foam mechanical behaviour.

### 3.5. Water Vapour Absorption Behaviour

**Figure 8:**
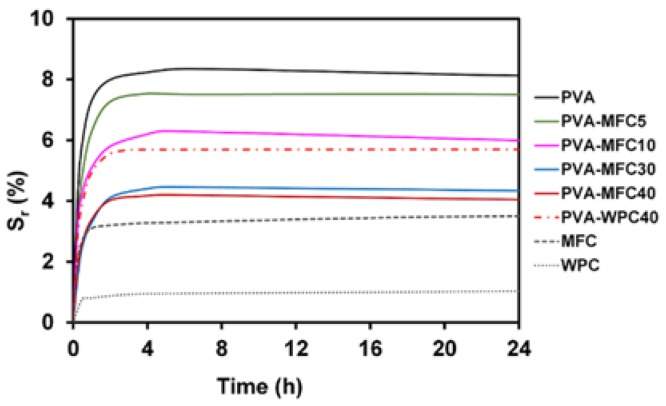
Swelling ratio of PVA-based foams.

The water vapour absorption behaviours of the neat PVA, PVA-MFC and PVA-WPC foams were investigated by determining the swelling ratio (*S*_r_). The trend of S_r_ versus time is reported in Figure 8, in which *S*_r_ values of WPC and MFC are included.

The results indicate that the addition of MFC reduces the affinity of PVA foams toward water, leading to a strong reduction in the swelling ratio. In particular, the addition of 5 wt % MFC induces a slight decrease of *S*_r_ value (about 7% with respect to the *S*_r_ value of neat PVA foam), while the addition of 30 and 40 wt % MFC decreases *S*_r_ by about 50%. WPC also affects the water absorption behaviour of PVA foams, but the recorded *S*_r_ decrease is lower than that obtained with the same amount of MFC, indicating that the difference in the swelling behaviour is mainly due to the increased interfacial area between MFC and the matrix.

## 4. Conclusions

Poly(vinyl alcohol) foams, with up to 40 wt % of microfibrillated cellulose, were successfully prepared by using a high-speed mixing and lyophilisation process. Foams exhibit a microcellular structure characterised by double porosity, evaluated following the water sublimation from the frozen PVA sample through a dynamic cryo-SEM experiment.

FTIR results indicated the formation of interactions via hydrogen bonding between hydroxyl groups located on cellulose fibres with those present along the polymer chains of PVA, resulting in an increase of the glass transition temperature and in a reduction of the affinity of PVA foams toward water. At low contents, MFC acts as a nucleating agent, while at higher loadings, MFC progressively inhibits PVA chain interactions, decreasing the overall crystallinity of the foam. Compared to the neat PVA foam, significant improvements in modulus and compression deflection were observed in PVA-MFC foams. Thus, the microfibrillated cellulose plays an important role for the overall improvement of the physical properties of PVA foams.

## Figures and Tables

**Table 1:** Thermal data of PVA-based foams.

	*T*_c_ (°C)	*T*_g_ (°C)	*T*_m_ (°C)	Δ*H*_m_ (J/g)	*X*_c_ (%)
PVA	119.9	42.9	176.8	25.1	15.5
PVA-MFC5	154.2	58.6	202.4	31.4	19.4
PVA-MFC10	149.2	56.6	200.8	30.3	18.8
PVA-MFC30	138.8	61.3	183.5	17.6	10.9
PVA-MFC40	127.8	63.3	166.2	11.2	6.9
PVA- WPC40	133.2	58.2	173.1	14.5	9.0

## Supplementary Materials

The following are available online at http://www.mdpi.com/2073-4360/10/8/813/s1.
